# The INSDC specifications—foundations for a FAIR and global INSDC

**DOI:** 10.1093/database/baag054

**Published:** 2026-09-07

**Authors:** Yuichi Kodama, Colman O’Cathail, Linda Yankie, the INSDC Implementation Committee, Alisha Ahamed, Takeshi Ara, Masanori Arita, Shelby L Bidwell, J Rodney Brister, Tony Burdett, Josephine Burgin, Vincent C Calhoun, Mark Cavanaugh, Jina Choi, Karen Clark, Guy Cochrane, Carla Cummins, Rajkumar Devaraj, Linda M Frisse, Takatomo Fujisawa, Dipayan Gupta, Maira Ihsan, Eugene Ivanov, Suran Jayathilaka, Ilene Karsch-Mizrachi, Ankur Lathi, Kyungbum Lee, Rasko Leinonen, Jun Mashima, Richard McVeigh, Terence D Murphy, Yasukazu Nakamura, Osamu Ogasawara, Toshihisa Okido, Christopher T O’Sullivan, Kim D Pruitt, Nadim Rahman, Valerie A Schneider, Adam Stine, Yasuhiro Tanizawa, Marianna Ventouratu, Zahra Waheed, Peter Woollard, David Yuan

**Affiliations:** Bioinformation and DDBJ Center, National Institute of Genetics, 1111 Yata, Mishima, Shizuoka 411-8540, Japan; European Molecular Biology Laboratory, European Bioinformatics Institute (EMBL-EBI), Wellcome Genome Campus, Hinxton, Cambridge, CB10 1SD, United Kingdom; National Center for Biotechnology Information, National Library of Medicine, National Institutes of Health, Bethesda, MD 20894, United States

## Abstract

Members of the International Nucleotide Sequence Database Collaboration (INSDC; https://www.insdc.org/) collect, exchange, and preserve comprehensive open nucleotide sequence information and provide tools for its access. The INSDC has stated its commitment to welcoming new members into the collaboration to be more representative of the global community of data and users. To reach this goal, a comprehensive definition of the INSDC data model and minimum requirements for data acceptance have been established. Here we describe the processes used to arrive upon these INSDC Specifications and lay out strategies for their continued upkeep to remain current and relevant.

**Database URL:**  https://www.insdc.org/

## Rationale and background

The International Nucleotide Sequence Database Collaboration (INSDC) was initially formed via an informal agreement to share data housed in the EMBL Data Library (now ENA, at the European Molecular Biology Laboratory’s European Bioinformatics Institute) in Europe, GenBank [originally housed at Los Alamos National Laboratory (now at the National Center for Biotechnology Information (NCBI), National Library of Medicine (NLM)] and the DNA Databank of Japan (DDBJ, at the National Institute of Genetics), promote open science, and support data sharing. It is now one of the longest-established and largest scientific data-sharing collaborations. However, in order to respond to the global nature of sequence data sharing, a more formalized collaboration was needed to facilitate the diversification and collaborative reach of the INSDC.

In 2023, the three founding members of INSDC signed a Founders Arrangement codifying the current collaboration and documenting the governance and foundational principles of the INSDC [1]. Part of the governance structure was the formal establishment of two committees within the collaboration: the Executive Committee (EC) to guide the strategic vision and the Implementation Committee (IC) to establish policies and procedures for the nucleotide sequence database to promote consistent data sharing. The EC recognized the need to formalize technical specifications within the collaboration and directed the IC to reconcile current data standards and validations with modern methods of data collection and usage. Historically, the implementation of specifications by each member has at times varied, on occasion causing confusion to database users. Thus, it has become essential to modernise and extend existing specifications of the data model, and formalise minimum requirements for data inclusion in INSDC databases. The additional governance body of the INSDC, the International Stakeholders Committee (ISC), was not involved in the process at this stage. However, the Executive Committee will endeavor to seek input from the ISC on the INSDC minimum specifications as and when this body meets.

Alongside this formalization of the partnership between the founding members, as well as the governance structure of the collaboration, the INSDC launched its Global Participation Initiative (https://www.insdc.org/global-participation/). The aims were to enable INSDC to broaden by including organizations or collaborators from around the world, build equitable systems for the global benefit from sequence data, and ensure diverse perspectives are represented in this important sequence data collaboration.

The prospect of inviting and onboarding new members is a new challenge for the nearly 40-year-old INSDC. Founding members, having grown their databases and services in parallel over lengthy timelines, have made incremental agreements on the mechanisms to achieve the goals of the collaboration. However, the result of this accumulation of agreements does not provide a suitable framing of overall specifications at the current point in time, as would be required by a future member looking to connect their database into the collaboration. Thus, a need was identified for the INSDC to modernise and consolidate existing INSDC Specifications into a coherent, implementation-agnostic framework to best describe the expectations of current and future members of the global sequence data exchange.

## Defining the INSDC data model

Historically, the primary technical specification used for data sharing throughout the collaboration was the INSDC Feature Table Definition (https://www.insdc.org/submitting-standards/feature-Table/), which details the format and requirements of the flat file, used traditionally for data exchange and display of sequence records. Changes in sequencing technology and data types required additional documentation and data models, such as the SRA data model and BioProject and BioSample schema [2, 3, 4]. However, as each Specification was developed, it was constructed as a singular topic and not written with an overall perspective of the breadth of data exchanged within the collaboration, nor with specific consideration to the relationship and interoperation between these data and metadata.

**Figure 1:**
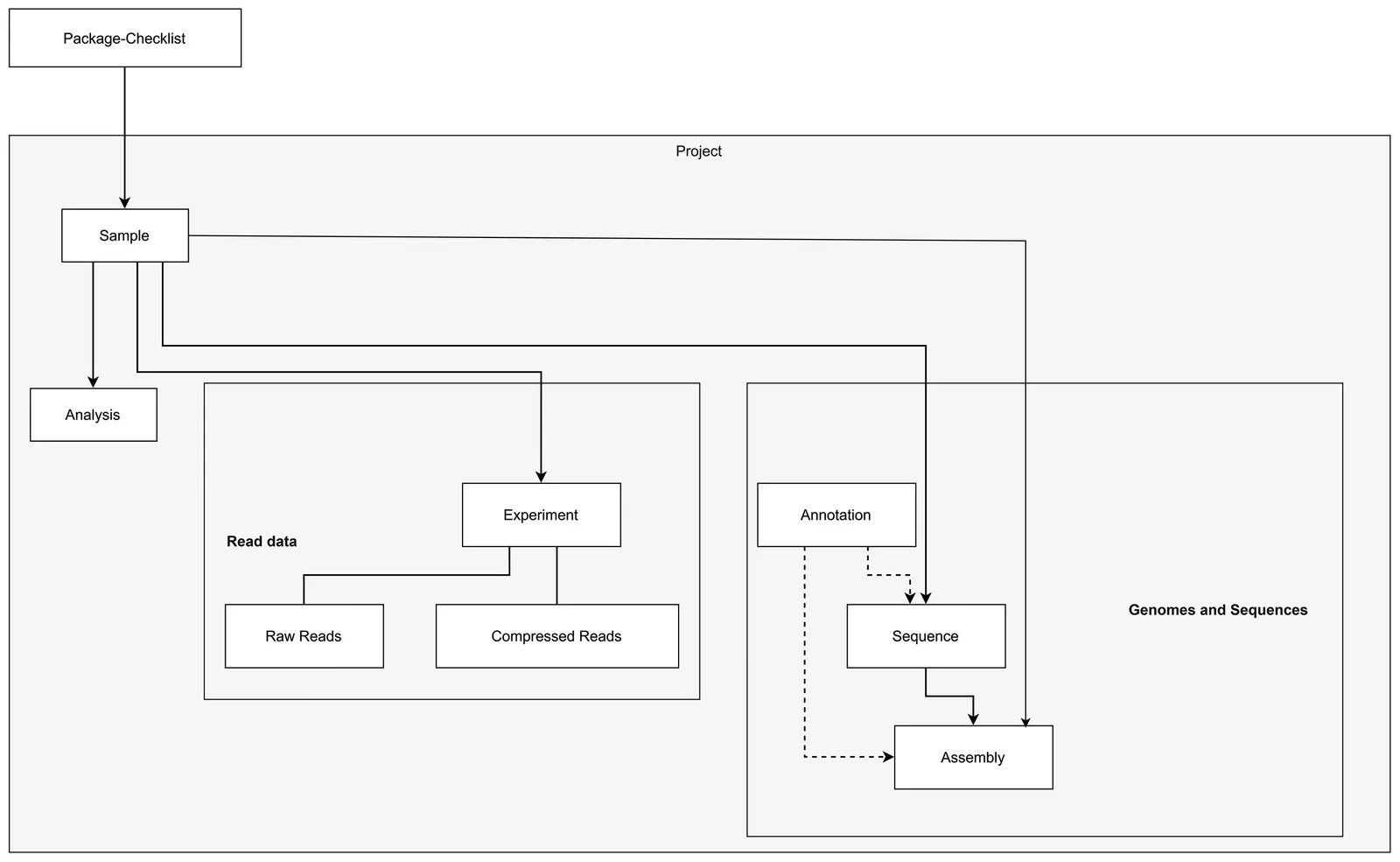
Data model an ideal diagram of how the INSDC minimal specifications could relate to one another. Most historical INSDC data will not follow this model to the letter, but this provides an optimal theoretical representation of the data. Dashed lines indicate optional relationships. Package-Checklist sits outside Project as it only has a relationship to Sample.

The first step in revising the Specifications was to categorize and define the data model into major types exchanged with an outlook to establishing precise standards for minimal validations for inclusion in the database. The broad data types ultimately determined to be in scope for enhanced standards documentation are: Project, Sample, Package-Checklist, Raw Reads, Compressed Reads, Experiment, Analysis, Assembled Sequence, Annotations and Assembly (Table 1). Figure 1 also shows an idealised model of how the data types can relate to one another.

**Table 1:** INSDC data types and definitions. The 10 broad data types in scope for the INSDC Specifications, with definitions describing the biological or technical content represented by each type.

Data type	Definition
Project	A collection of biological data and description of research effort related to a single initiative, originating from a single organization or from a consortium
Sample	The description of the biological source materials (including how and where they were obtained) used in experimental assays
Package-Checklist	A predetermined set of metadata requirements for sample registration based on the type of biological sample
Raw Reads	Sequence read files containing base-level information
Compressed Reads	Sequence read files without base-level qualities
Experiment	The description of sequencing experimental information, including methods, platform, library and machine configuration
Analysis	Secondary data files which are derived from computational workflows or provide supplemental biological context to INSDC nucleotide data
Sequence	Nucleotide sequences assembled in biological order from individual reads
Annotations	Description and location of the biological features in the cited sequence
Assembly	Description of the set of assembled sequences and related metadata, that identifies the chromosomes, unlocalized sequences and unplaced sequences that represent a genome

## Establishing the INSDC specifications

With the data types established, the IC assigned *ad hoc* committees with two representatives from each member node. The chairing of these committees was shared across the members and regular meeting schedules were established with the ultimate goal of documenting the agreed Specification for each data type. The committees’ work focused on the establishment of Specifications while allowing each member flexibility to determine implementation within their own systems. Although each of the committees met separately, the complete corpus of data was considered in each discussion and committee membership often overlapped.

A structured reporting template was adopted to focus discussion and promote consistency between the separate committees. The form was designed to emphasize the needs of current and future database users and encourage data-driven design decisions. Along with a section for validation requirements, committees considered data use cases and publicly available validation tools that could aid data submitters in preparing valid submissions. Discussions of data exchange formats, data availability across INSDC members, and standards for cross-referencing other data types were also incorporated into these reports with an eye to increasing interoperability between the data types and providing complex content in a meaningful way to address user needs. Discussions also addressed interoperability beyond the INSDC itself, recognising that INSDC accessions serve as primary identifiers within the wider biodata ecosystem. Resources such as UniProt, Ensembl, and RefSeq, depend on stable, well-described INSDC records to maintain consistent cross-resource linkage and provenance

The drafts were presented at a series of joint IC-EC meetings that provided an open forum in which representatives could ask questions and discuss any complications with committee members. These conversations focused on the Specifications and the validation required for data to conform, acknowledging that further dialog is needed regarding the future of data exchange formats. A vote was then held to accept each Specification or send back to the committee to address specific concerns. A comprehensive review of current documentation and implementation protocols by each member was then undertaken to resolve any discrepancies with the proposed new Specifications.

The agreed final Specifications define the minimal validation threshold required for data to be accepted and shared under INSDC. No INSDC database will accept data that fails to conform to the relevant Specifications. Data submitters should expect that conforming to the relevant Specifications for their data, alongside compliance with relevant requirements laid out in the respective database’s terms of use, will be sufficient for acceptance into INSDC. However, individual members may offer more extensive validation procedures to support data that offer higher or deeper levels of annotation and quality. Additional validations imposed by individual INSDC members will be publicly communicated.

The INSDC Specifications themselves are not presented in detail in this paper since they are expected to evolve over time in response to user needs. Instead, the most up-to-date version of the Specifications is available on the INSDC website at: https://www.insdc.org/insdc-minimal-specifications/, on GitHub at: https://github.com/insdc/minimum-specifcations and will be made available via the websites of all three founder members. The Specifications will be versioned, and all versions will be available on GitHub. At the time of writing, all specifications are version 1.0, and snapshotted as a release on GitHub to preserve the state at the time of publication of this manuscript: https://github.com/insdc/minimum-specifcations/releases/tag/2026_Publication.

## Future outlook

The creation of comprehensive INSDC Specifications provides transparency to data submitters, database users, and future INSDC members.

A critical aspect of the Specifications is flexibility. The Specifications exist in living documents that can be updated to reflect the ever-changing landscape of biological science and data collection and analysis. Additional requirements and validations may be applied in the future and will be documented clearly within the Specifications framework. In addition to minimum standards for acceptance, best practices may also be recommended to enhance quality and consistency. Highlighting data that has been validated beyond the INSDC Specifications would also increase the reusability of data.

A key to the success of the INSDC is the ongoing data sharing among its members. The next step in formalizing the Specifications is to codify the details of the data exchange across members, including any potential new members. For each data type, the timing of exchange, availability status definitions, and timely adherence to those definitions will be delineated. Also, the precise formats used for exchange will be determined in a manner that promotes consistency and ease of exchange, while allowing each member flexibility in the technical implementation to achieve the agreed-upon format. The INSDC also maintains active engagement with external standards bodies, including the Global Alliance for Genomics and Health (GA4GH) and the Genome Standards Consortium (GSC), both shaping and drawing on global standards development to further the objectives of FAIR, open sequence data sharing.

The formal establishment of data types and documentation of minimal validation supports INSDC’s commitment to open data and global representation. This foundation is key to the continued success of the collaboration in its mission to provide free and comprehensive nucleotide sequence data to the scientific community to support fields from infectious disease, through biotechnology, to biodiversity.
